# Modulation of the Neuro–Cancer Connection by Metabolites of Gut Microbiota

**DOI:** 10.3390/biom15020270

**Published:** 2025-02-12

**Authors:** Alice N. Mafe, Dietrich Büsselberg

**Affiliations:** 1 Department of Biological Sciences, Faculty of Sciences, Taraba State University, Main Campus, Jalingo 660101, Taraba State, Nigeria; mafealice1991@gmail.com; 2 Weill Cornell Medicine-Qatar, Education City, Qatar Foundation, Doha Metropolitan Area, Doha P.O. Box 22104, Qatar

**Keywords:** gut microbiota, neuro–cancer connection, microbial metabolites, gut–brain axis and immune modulation

## Abstract

The gut–brain–cancer axis represents a novel and intricate connection between the gut microbiota, neurobiology, and cancer progression. Recent advances have accentuated the significant role of gut microbiota metabolites in modulating systemic processes that influence both brain health and tumorigenesis. This paper explores the emerging concept of metabolite-mediated modulation within the gut–brain–cancer connection, focusing on key metabolites such as short-chain fatty acids (SCFAs), tryptophan derivatives, secondary bile acids, and lipopolysaccharides (LPS). While the gut microbiota’s impact on immune regulation, neuroinflammation, and tumor development is well established, gaps remain in grasping how specific metabolites contribute to neuro–cancer interactions. We discuss novel metabolites with potential implications for neurobiology and cancer, such as indoles and polyamines, which have yet to be extensively studied. Furthermore, we review preclinical and clinical evidence linking gut dysbiosis, altered metabolite profiles, and brain tumors, showcasing limitations and research gaps, particularly in human longitudinal studies. Case studies investigating microbiota-based interventions, including dietary changes, fecal microbiota transplantation, and probiotics, demonstrate promise but also indicate hurdles in translating these findings to clinical cancer therapies. This paper concludes with a call for standardized multi-omics approaches and bi-directional research frameworks integrating microbiome, neuroscience, and oncology to develop personalized therapeutic strategies for neuro-cancer patients.

## 1. Introduction

The intricate relationship between the gut, brain, and various systemic processes has garnered significant attention recently, as has increased interest in the gut–brain axis (GBA), which traditionally refers to the bidirectional communication between the gut and the brain [1]. The GBA represents a bidirectional communication network encompassing the central nervous system (CNS), the enteric nervous system (ENS), and the gut microbiota [2]. This dynamic axis is pivotal in maintaining homeostasis and influencing neurodevelopment, immune function, and metabolic regulation [3]. Disruptions in the GBA have been implicated in various pathological conditions, including neurodegenerative diseases, psychiatric disorders, and cancers [4]. Emerging evidence has established a direct link between the gut–brain axis and cancer, particularly brain cancer [5]. The gut microbiota plays a crucial role in modulating systemic inflammation, immune responses, and metabolic pathways, all of which contribute to tumorigenesis in the brain [6]. Dysbiosis, characterized by an imbalance in gut microbial composition, has been associated with increased systemic inflammation and blood–brain barrier permeability, facilitating the progression of brain cancer [7]. Gut microbiota-derived metabolites, including SCFAs and tryptophan derivatives, have been shown to influence neuroinflammation and immune surveillance, potentially affecting the tumor microenvironment in the brain [8]. Equally, gut dysbiosis may impair anti-tumor immunity by altering the balance of regulatory and pro-inflammatory immune cells, thereby promoting brain tumor progression [9]. Furthermore, recent studies have demonstrated that gut microbiota can impact the efficacy of brain cancer therapies, including immunotherapy and chemotherapy [10]. Certain bacterial species enhance the responsiveness to immune checkpoint inhibitors, while others contribute to resistance by modulating host immune pathways [11]. This evidence underscores the significance of gut microbiota in shaping the tumor microenvironment and therapeutic outcomes in brain cancer [12]. Given these findings, targeting the gut–brain axis through microbiome-based interventions presents a promising avenue for improving brain cancer treatment strategies.

A key player in this axis is the gut microbiota, a diverse microbial ecosystem producing a myriad of metabolites that modulate systemic processes [13]. These metabolites, such SCFAs, tryptophan derivatives, and secondary bile acids, act as molecular messengers, influencing cellular pathways critical to both neurobiology and cancer progression [14]. For instance, SCFAs like butyrate have demonstrated neuroprotective and anti-inflammatory properties, while tryptophan metabolites can mediate immune escape mechanisms in tumors [15]. Despite these individual insights, the potential interplay between gut microbiota metabolites, brain function, and cancer remains underexplored [16]. The novelty of this study lies in resolving a critical gap in the literature: the lack of integrated investigations into how gut microbiota-derived metabolites simultaneously impact the brain and cancer. This paper focuses explicitly on brain cancer, emphasizing the gut–brain–cancer axis and highlighting the intricate interactions between gut microbiota, neurobiology, and oncogenesis in the brain. By focusing on this triad, this work aims to unravel novel mechanisms, shed light on emerging therapeutic possibilities, and pave the way for interdisciplinary research bridging neurobiology, oncology, and microbiology. This synthesis points out the systemic significance of gut microbiota metabolites and marks the need for targeted research in this promising yet underdeveloped field.

## 2. Overview of the Gut Microbiota and Its Metabolites

The gut microbiota, a dynamic and complex community of microorganisms residing in the gastrointestinal tract, plays a pivotal role in shaping human physiology and health [6]. Beyond its well-established digestion and nutrient absorption functions, the microbiome profoundly affects systemic processes by producing bioactive metabolites. These microbial metabolites act as molecular signals, orchestrating interactions between the gut, immune system, brain, and other organs [17].

In the context of the gut–brain–cancer axis, recognizing the diversity of the gut microbiota and the spectrum of metabolites they produce is essential [18]. While some metabolites, such SCFAs and tryptophan derivatives, have been extensively studied for their beneficial and pathological roles, emerging metabolites with dual neuro–cancer relevance remain poorly characterized [19]. Emerging metabolites such as phenolics and sphingolipids are gaining attention for their roles in neurobiology and cancer development [20]. Phenolic compounds, derived from dietary sources and microbial metabolism, exhibit potent antioxidant, anti-inflammatory, and immunomodulatory properties [21]. These metabolites influence brain function by modulating neurotransmitter systems, reducing oxidative stress, and regulating neuroinflammation, which are crucial in neurodegenerative diseases and brain tumors [22]. Also, phenolics can interfere with cancer cell proliferation by targeting signaling pathways like NF-κB and PI3K/Akt, thereby inhibiting tumorigenesis [23]. Their ability to cross the blood–brain barrier further underscores their significance in neuro-oncological interactions, suggesting a potential therapeutic role in neurodegenerative conditions and brain cancers [24]. On the other hand, Sphingolipids are bioactive lipids that play essential roles in cell signaling, apoptosis, and membrane integrity [25]. These metabolites are critical regulators of neurodevelopment and brain homeostasis, but their dysregulation is implicated in neurodegenerative diseases and brain cancer [26]. Sphingolipid metabolism influences neural inflammation, synaptic function, and myelination, linking them to disorders like Alzheimer’s and Parkinson’s disease [27]. In cancer, sphingolipids such as ceramides and sphingosine-1-phosphate (S1P) are key mediators of tumor progression, angiogenesis, and metastasis [28]. While ceramides promote apoptosis, S1P fosters tumor cell survival and immune evasion, creating a dynamic balance that affects neuro-oncological outcomes [29]. The intricate interplay between sphingolipid metabolism, neuroinflammation, and tumorigenesis highlights their dual role as potential biomarkers and therapeutic targets in neuro-cancer research [30]. The growing recognition of phenolics and sphingolipids in the gut–brain–cancer axis emphasizes their importance in understanding the metabolic underpinnings of glioblastoma [31]. Their independent yet interconnected roles suggest novel intervention strategies that harness these metabolites for neuroprotection and cancer therapy. Future research exploring their mechanisms and therapeutic modulation could pave the way for innovative treatments targeting metabolic pathways involved in neurological disorders and tumorigenesis. This section examines the gut microbiota’s metabolic capabilities, exploring well-known and novel compounds with neurobiology and cancer progression. Through this lens, we aim to elucidate the microbiome’s potential as a therapeutic target and a key modulator of the gut–brain–cancer connection

### 2.1. Diversity of Gut Microbiota and Key Genera Involved in Metabolite Production

The gut microbiota is one of the most diverse and densely populated microbial ecosystems in the human body, consisting of trillions of microorganisms, including bacteria, archaea, fungi, viruses, and protozoa [32]. This ecosystem exhibits significant inter-individual variability influenced by diet, genetics, age, and geographical location [33]. However, common bacterial phyla dominate the gut microbiota, including *Firmicutes*, *Bacteroidetes*, *Actinobacteria*, and *Proteobacteria*. Within this diverse community, specific genera stand out for their pivotal role in metabolite production [34]. For instance, *Lactobacillus*, a genus under the phylum Firmicutes, is renowned for producing lactic acid and contributing to synthesizing SCFAs [35]. It is also instrumental in generating bioactive compounds, such as bacteriocins and indoles, with immunomodulatory and neuroprotective properties [36]. Similarly, *Bacteroides* sp., a dominant group under the phylum *Bacteroidetes*, are key players in the breakdown of complex polysaccharides, producing SCFAs like propionate and acetate that regulate gut health and systemic inflammation [37]. Other notable contributors include *Clostridium* sp., *Faecalibacterium* sp., and *Akkermansia* sp., which have specialized roles in metabolite synthesis [38]. For example, *Faecalibacterium prausnitzii* is a significant producer of butyrate, a metabolite with anti-inflammatory and epigenetic modulation effects [39]. Meanwhile, *Akkermansia muciniphila* degrades mucins to produce propionate and acetate, metabolites linked to improved gut barrier integrity and immune regulation [40]. This diversity and metabolic functionality denote the importance of gut microbiota as a metabolic powerhouse, influencing gastrointestinal health and systemic and neurological outcomes [41]. By investigating these key genera, we gain insights into the microbiome’s capacity to modulate the gut–brain–cancer axis, paving the way for targeted microbiome-based interventions.

### 2.2. Key Metabolites: SCFAs, Tryptophan Metabolites, Secondary Bile Acids, and LPS

The gut microbiota synthesizes a vast array of metabolites that serve as critical mediators in host–microbe interactions [42]. These metabolites influence diverse biological processes, including inflammation, immune modulation, and communication along the gut–brain axis [43]. Among these, short-chain fatty acids (SCFAs), tryptophan metabolites, secondary bile acids, and LPS stand out for their established and emerging roles in the neuro–cancer connection [44] as follows:

• SCFAs, primarily acetate, propionate, and butyrate, are fermentation products of dietary fibers by gut bacteria such as *Faecalibacterium* sp. [45]. Butyrate is a crucial energy source for colonocytes and has potent anti-inflammatory properties. It strengthens gut barrier integrity by regulating tight junction proteins and reducing gut permeability [46]. SCFAs also influence systemic inflammation, immune cell differentiation, and epigenetic modulation through histone deacetylase (HDAC) inhibition [47]. Their neuroprotective effects include modulating neurotransmitter production and reducing neuroinflammation, making SCFAs central to the gut–brain–cancer axis [48];
• Tryptophan metabolites: Tryptophan, an essential amino acid, undergoes metabolism by gut microbiota into bioactive compounds such as indoles, serotonin, and kynurenine. Indoles, produced by bacteria like *Lactobacillus* sp., have been linked to immune regulation, epithelial barrier maintenance, and signaling within the gut–brain axis [49]. Serotonin, a neurotransmitter synthesized partly in the gut, influences mood and gastrointestinal motility [50]. Kynurenine, derived from host-mediated tryptophan metabolism, has dual roles in immunosuppression and neurodegeneration [51]. The interplay of these metabolites with cancer progression and brain function presents a fertile ground for research into therapeutic interventions [52];
• Secondary bile acids: Gut microbiota converts primary bile acids into secondary bile acids, such as deoxycholic acid (DCA) and lithocholic acid (LCA), through deconjugation and transformation processes [53]. These metabolites influence lipid metabolism, immune signaling, and gut microbiota composition [54]. However, secondary bile acids also exhibit context-dependent roles in cancer [55]. While some promote carcinogenesis by inducing DNA damage and inflammation, others inhibit tumor growth by modulating apoptosis and cellular differentiation [56]. Their impact on neuroinflammation and systemic signaling underscores their significance in the gut–brain–cancer triad [57];
• LPS. a structural component of gram-negative bacterial membranes [58], acts as a potent endotoxin [59]. The translocation of LPS into the systemic circulation due to increased gut permeability triggers chronic inflammation through toll-like receptor 4 (TLR4) activation [60]. This inflammation is implicated in cancer progression, particularly in fostering a pro-tumorigenic microenvironment [61]. In addition, LPS-induced inflammation has been linked to neurodegenerative conditions, emphasizing its dual impact on cancer and neurological health [62].

Integrating these metabolite pathways reveals the complex and interconnected roles of the gut microbiota in modulating host health. While the established roles of SCFAs, tryptophan metabolites, secondary bile acids, and LPS are well documented, further exploration into their specific contributions to the neuro–cancer connection could uncover novel therapeutic targets.

### 2.3. Novelty: Emerging Metabolites with Neuro-Cancer Implications

Recent research spotlights an array of gut microbiota-derived metabolites that exhibit promising, yet underexplored, roles in the neuro–cancer connection [63]. Background research has laid the foundation for understanding how these metabolites influence various physiological processes, particularly within the context of brain function and cancer development [64]. It has been shown that gut microbiota metabolites, such as indoles and polyamines, are not only involved in maintaining intestinal homeostasis but also in modulating brain activity and immune responses [65]. These compounds are increasingly recognized for their potential to affect both brain health and cancer biology, influencing neuroinflammation, neurotransmitter balance, and tumor progression [66]. Among these, indoles and polyamines stand out as emerging candidates with significant potential to influence both brain health and cancer biology [67]. Indoles, derived from tryptophan metabolism, are known to affect neural signaling and inflammation, while polyamines, which are involved in cell proliferation and differentiation, may play key roles in tumor growth and neuroprotection [68]. Their unique mechanisms of action and dual roles in modulating neurobiological and oncological pathways make them compelling subjects for future investigations. Understanding how these metabolites intersect with brain and cancer biology will be essential for uncovering novel therapeutic strategies in neuro-oncology and neurodegenerative diseases.

• Indoles: Indoles are metabolic byproducts of tryptophan catabolism by gut bacteria such as *Clostridium* sp., *Escherichia coli*, and *Lactobacillus* sp. [69]. These metabolites are pivotal in maintaining gut epithelial integrity by enhancing tight junction expression and reducing gut permeability [70]. Beyond their localized effects in the gut, indoles signal systemically through the aryl hydrocarbon receptor (AhR), influencing immune regulation and inflammation [71]. In neurobiology, indoles have been shown to modulate serotonin production, a key neurotransmitter in mood regulation and cognitive function [72]. They also exhibit antioxidant and anti-inflammatory properties that could mitigate neurodegenerative processes [73]. In oncology, indole derivatives like indole-3-carbinol (I3C) have demonstrated anti-cancer potential by inducing apoptosis, suppressing cell proliferation, and modulating estrogen metabolism in hormone-responsive cancers [74]. However, the precise interplay of indoles in the neuro–cancer axis remains largely uncharted, warranting further investigation into their systemic and localized effects [75];
• Polyamines: Polyamines, including putrescine, spermidine, and spermine, are small, positively charged molecules produced by gut microbiota and host cells [76]. These metabolites are integral to cellular functions such as DNA stabilization, protein synthesis, and cell proliferation [77]. Evidence shows that polyamines are primarily generated by certain bacteria in the gut, including *Lactobacillus* sp., *Bifidobacterium* sp., and *Enterococcus* species [78]. These bacteria metabolize dietary components to produce polyamines [79]. While polyamines are essential for normal cellular function, their dysregulation is implicated in both cancer progression and neurodegenerative diseases [80]. In cancer, elevated polyamine levels support tumor growth by promoting angiogenesis, evading apoptosis, and enhancing cellular proliferation [81]. Conversely, some studies suggest that polyamines like spermidine exhibit anti-aging and neuroprotective effects, potentially modulating autophagy and reducing oxidative stress in the brain [82]. This duality articulates the need to investigate their context-dependent roles in cancer and neurological health, especially in the gut–brain–cancer axis;
• Uncharted territory and research opportunities: The intersection of indoles and polyamines with the neuro–cancer connection is a nascent field with numerous unanswered questions [83]. As summarized in Table 1, gut microbiota-derived metabolites play crucial roles in modulating neurobiological processes and cancer pathways. This table features key metabolites and their effects on both the brain and cancer and identifies current knowledge gaps, with data compiled from recent studies on gut–brain and gut–cancer interactions.

By focusing on these underexplored metabolites, researchers can uncover novel mechanisms underlying the gut microbiota’s role in systemic health. Such efforts not only address current gaps in knowledge but also offer the potential to develop innovative interventions for diseases at the intersection of cancer and neurobiology.

## 3. Mechanisms Linking Gut Microbiota Metabolites to Neurobiology and Cancer

The gut microbiota, through its complex metabolic activities, influences a wide array of physiological processes, including neurobiology and cancer progression [90]. Recent advances have revealed that metabolites produced by gut microorganisms are not merely byproducts of digestion but active signaling molecules that can impact distant organs, including the brain and tumors [91]. These metabolites mediate a range of interactions via direct and indirect mechanisms, influencing the immune system, inflammatory pathways, and cellular processes essential for brain function and cancer cell behavior [92]. Comprehending the pathways through which these metabolites act is crucial to unraveling the gut–brain–cancer axis. This section delves into the various mechanisms by which gut microbiota-derived metabolites influence neurobiology and cancer [93]. It explores how these metabolites cross the blood–brain barrier, modulate neuroinflammation, and impact neurotransmitter production in the brain. Equally, it examines their role in cancer progression, including effects on tumor microenvironments, immune responses, and cancer cell proliferation [94]. Depending on the context, these complex and multifaceted interactions reveal protective and harmful roles. Therefore, studying these mechanisms is essential for potential therapeutic strategies targeting the gut–brain–cancer connection.

### 3.1. Modulation of the Immune System via SCFAs (Butyrate’s Role in Tregs and Anti-Inflammatory Pathways)

SCFAs, particularly butyrate, are produced by gut microbiota during the fermentation of dietary fibers and play a significant role in modulating the immune system [95]. Butyrate, a key SCFA, has garnered attention due to its ability to influence local immune responses in the gut and systemic immunity, including neuroinflammation and cancer immunity [96]. One of the central mechanisms through which butyrate modulates the immune system is by promoting the differentiation and function of regulatory T cells (Tregs), a subset of immune cells known for their role in maintaining immune tolerance and preventing excessive inflammation [97]. Butyrate influences the epigenetic regulation of Tregs by inhibiting histone deacetylases (HDACs), which enhances the expression of genes essential for Treg development and function [98]. This action helps to maintain immune homeostasis and suppresses the activation of pro-inflammatory immune responses that could otherwise lead to autoimmune diseases or chronic inflammatory conditions, including neuroinflammation and cancer-related inflammation [99].

In addition to its role in Treg differentiation, butyrate also plays a critical role in regulating anti-inflammatory pathways [100]. It can enhance the production of anti-inflammatory cytokines, such as interleukin-10 (IL-10), and inhibit the secretion of pro-inflammatory cytokines like tumor necrosis factor-alpha (TNF-α) and interleukin-6 (IL-6). This anti-inflammatory profile is crucial for maintaining brain health, as excessive neuroinflammation is linked to neurodegenerative diseases, including Alzheimer’s and Parkinson’s disease, as well as the progression of certain cancers [101]. Butyrate’s influence on the microbiota–gut–brain axis emphasizes the role of the gut microbiota in this communication, which affects the brain’s immune environment, potentially altering neuroinflammation and even modulating cancer-related pathways [102]. Butyrate’s effects on the immune system are especially pertinent in cancer, where tumor-promoting inflammation can influence tumor growth, metastasis, and immune evasion [103]. By promoting a balanced immune response by activating Tregs and suppressing inflammatory mediators, butyrate can act as a natural immune modulator, potentially offering therapeutic benefits in cancer treatment by improving anti-tumor immunity and reducing tumor-induced inflammation [104]. However, while the immunomodulatory effects of butyrate are well documented, further research is needed to understand better the intricate balance between its anti-inflammatory actions and potential roles in other immune-mediated processes, including cancer immunosurveillance and neuroimmune interactions [105].

### 3.2. Tryptophan–Kynurenine Pathway: Neurotoxicity and Tumor Immune Escape

The tryptophan–kynurenine pathway is one of the key metabolic routes through which gut microbiota influence neurobiology and cancer [106]. Tryptophan, an essential amino acid, is metabolized by the gut microbiota and host enzymes into a range of metabolites, with kynurenine being the most prominent intermediate [107]. This pathway has been widely studied due to its significant effects on immune modulation, neurobiology, and cancer progression [108]. One of the most notable aspects of the tryptophan-kynurenine pathway is its role in regulating immune responses [109]. The accumulation of kynurenine and its downstream metabolites can lead to the activation of the AhR, a transcription factor involved in regulating immune cell differentiation, including the development of regulatory T cells (Tregs) [110]. This mechanism can contribute to tumor immune escape, as the kynurenine-mediated activation of Tregs may suppress anti-tumor immune responses [111]. This immunosuppressive effect creates a tumor-friendly microenvironment, facilitating tumor growth and metastasis [112].

**Figure 1:**
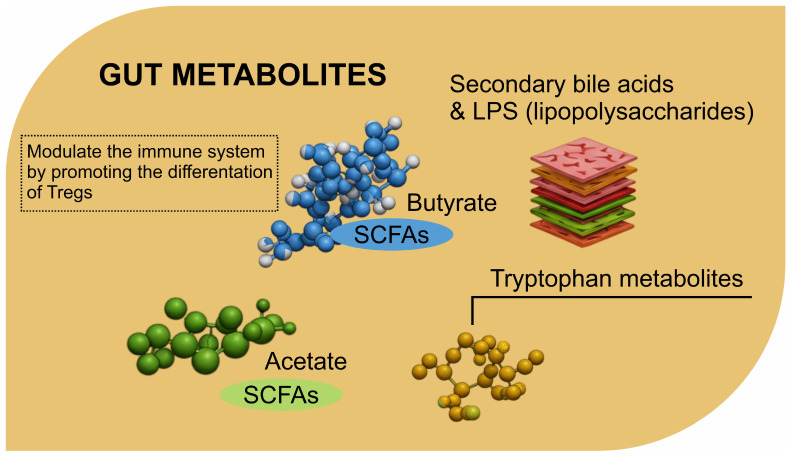
Gut Metabolite in the gut (created using BioRenderhttps://app.biorender.com/user/signin).

**Figure 2:**
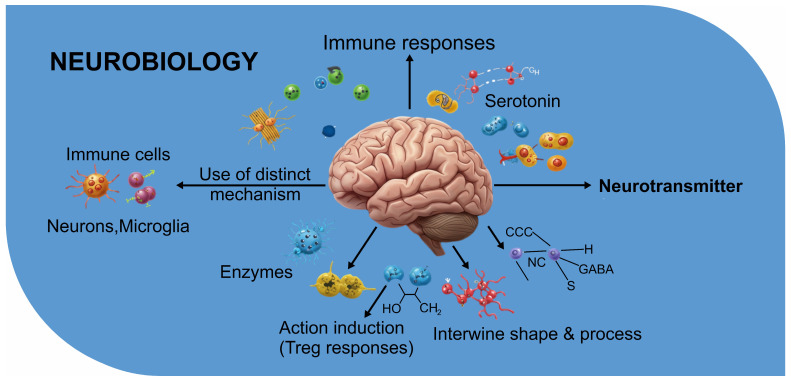
Neurobiology in the brain (created using BioRender).

**Figure 3:**
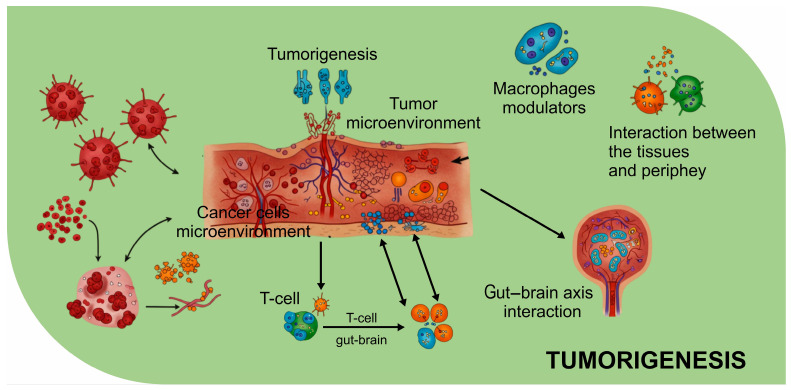
Tumorigenesis in the brain (created using BioRender).

On the neurobiology front, the kynurenine pathway is also implicated in neurotoxicity and neuroinflammation [113]. The metabolites of kynurenine, such as quinolinic acid, are neurotoxic and have been linked to various neurodegenerative diseases, including Alzheimer’s and Parkinson’s diseases [114]. These neurotoxic metabolites can cross the blood–brain barrier and lead to excitotoxicity, which damages neurons and exacerbates neuroinflammatory responses [115]. The interplay between kynurenine metabolites and the brain’s immune system makes this pathway a critical bridge between gut-derived metabolites, neuroinflammation, and cancer progression [116]. Moreover, the kynurenine pathway is a vital link between the gut microbiota and cancer immune escape [117]. Tumors can manipulate this pathway to enhance the production of immunosuppressive metabolites, which help create an environment where cancer cells can evade immune surveillance [118]. This pathway’s influence on neuroinflammation and tumor immunity elevates the potential of targeting the tryptophan–kynurenine axis as a therapeutic approach for diseases involving both the brain and cancer, including brain tumors [119]. Figure 1 illustrates the role of gut metabolites in modulating host physiology, particularly in influencing immune responses and systemic inflammation. Independently, Figure 2 highlights the neurobiological mechanisms that interact with metabolic signals, demonstrating how microbial metabolites can influence neurotransmitter synthesis and neuroinflammation. Meanwhile, Figure 3 presents tumorigenesis as a multifactorial process, where both gut-derived metabolites and neurobiological factors contribute to cancer progression. These mechanisms do not occur sequentially but function as independent yet interconnected events, where gut metabolites (Figure 1) shape systemic immune responses, neurobiological pathways (Figure 2) mediate stress and inflammation, and tumorigenesis (Figure 3) emerge from the cumulative effects of these interactions. This complex network suggests a bidirectional influence among gut metabolism, neurological processes, and cancer development rather than a linear causative pathway.

### 3.3. Novelty: Lack of In Vivo Data Connecting Polyamine Metabolism to Neuro–Cancer Crosstalk

Polyamines, such as putrescine, spermidine, and spermine, are small organic molecules produced by gut microbiota and host cells [120]. In addition to gut bacteria, host cells, particularly those in the intestinal epithelium and other tissues, also contribute to polyamine production, synthesizing these metabolites through the enzyme-mediated decarboxylation of amino acids such as ornithine and methionine [121]. These polyamines play essential roles in cellular growth, differentiation, and survival, making them critical for neurobiology and cancer biology [122]. However, despite their importance, there remains a significant gap in the literature regarding the in vivo data connecting polyamine metabolism to the crosstalk between the gut microbiota, neurobiology, and cancer [123]. The novelty in this area is that polyamine metabolism has not been thoroughly explored in brain function and cancer progression, especially for their interaction with gut-derived metabolites [124]. Polyamines affect cell proliferation and apoptosis, making them highly relevant for tumorigenesis. In the brain, they influence neurotransmitter synthesis, neuronal survival, and synaptic plasticity [122]. However, how polyamines derived from gut microbiota interact with brain function and tumor growth remains largely underexplored [125].

Polyamines also modulate immune responses, and their dysregulation can lead to immune escape mechanisms in tumors, promoting cancer progression [126]. However, there is limited cognizance of how alterations in polyamine metabolism, driven by the gut microbiota, impact the immune system in both the brain and tumor microenvironment [127]. Recent studies suggest that polyamines may affect the balance between pro-inflammatory and anti-inflammatory responses. Still, more in vivo data are needed to establish the direct connections between polyamine metabolism and neuro–cancer crosstalk [81]. The lack of extensive in vivo studies in this field opens up an exciting area of research that could significantly enhance our awareness of how gut microbiota-derived metabolites influence the microbiota–gut–brain–cancer axis and introduce the additional complexity of the microbiota’s involvement in brain cancer modulation [128]. Investigating polyamine metabolism in vivo could offer new therapeutic targets for cancers, particularly brain tumors, where the gut–brain–cancer interaction is still under investigation [129].

## 4. Emerging Evidence from Preclinical and Clinical Studies

The growing recognition of the gut–brain–cancer axis has sparked considerable interest in exploring how metabolites produced by gut microbiota influence neurobiology and cancer progression [130]. While much of the existing literature has focused on the mechanistic recognition of these interactions, recent preclinical and clinical studies are beginning to uncover compelling evidence supporting the involvement of gut microbiota-derived metabolites in both neurological disorders and cancer [22]. These studies offer insights into the therapeutic potential of targeting the gut microbiota and its metabolites to modulate cancer progression, immune responses, and neuroinflammation [131]. This section reviews the emerging preclinical and clinical evidence that connects specific metabolites, such as SCFAs, tryptophan metabolites, and polyamines, to the regulating neurobiological functions and cancer biology. By synthesizing data from animal models, human clinical trials, and observational studies, we call attention to how the gut microbiota influences tumor growth, distant metastasis, and neuroimmune interactions. Plus, this section aims to identify key findings that point to potential therapeutic strategies leveraging gut microbiota modulation to benefit cancer treatment, neurological health, and overall systemic immunity. Despite these promising advances, difficulties remain, particularly in translating these findings into clinical applications. Further studies, especially those utilizing in vivo models and larger patient cohorts, are needed to understand the complexities of the gut–brain–cancer interaction better and validate the potential of microbiome-based interventions in cancer and neurodegenerative diseases.

### 4.1. Preclinical Studies: Rodent Models of Gut Dysbiosis Demonstrating Neurobehavioral and Tumorigenic Outcomes

Preclinical studies using rodent models have provided valuable insights into how gut dysbiosis, the imbalance in gut microbiota, can influence neurobehavioral outcomes and tumorigenesis [132]. In particular, studies focusing on rodents with altered gut microbiota have demonstrated significant changes in behavior, cognition, and susceptibility to cancer [133]. These models allow researchers to explore the intricate relationships between the gut microbiota, the brain, and cancer, offering a foundation for future therapeutic interventions [5]. Rodent studies of gut dysbiosis have shown that shifts in gut microbial composition can lead to changes in BBB permeability, neuroinflammation, and even behavioral deficits, such as anxiety, depression, and cognitive impairments [134]. For instance, the depletion of beneficial bacteria like *Lactobacillus* and *Bifidobacterium* has been associated with increased levels of pro-inflammatory cytokines in the brain, contributing to neuroinflammation and changes in neural function [135]. Similarly, gut microbiota alterations have been linked to the activation of neuroimmune pathways, influencing brain health and the development of neurodegenerative diseases [136]. Regarding tumorigenesis, studies on rodent models with gut dysbiosis have revealed that specific microbial imbalances can promote cancer progression [137].

For example, microbiota alterations have been associated with an increased incidence of glioblastoma and other tumor types [138]. In these models, gut-derived metabolites, such as SCFAs and bile acids, can modulate immune responses, influence tumor microenvironments, and enhance cancer cell proliferation [63]. Notably, the ability of gut-derived metabolites to impact the immune system by modulating Treg cells and promoting an anti-inflammatory state suggests a possible mechanism by which dysbiosis may enable tumor growth and immune escape [139]. These preclinical findings provide crucial evidence that gut dysbiosis may catalyze neurological and cancerous alterations [140]. They convey the need for further investigation into microbiome-based therapies that can potentially mitigate neurobehavioral and tumorigenic outcomes by restoring a healthy gut microbiota.

### 4.2. Clinical Studies: Correlations Between Gut Dysbiosis, Altered Metabolite Profiles, and Brain Tumors

In recent years, clinical studies have begun to explore the relationship between gut dysbiosis, altered metabolite profiles, and brain tumors, particularly glioblastoma, one of the most aggressive and treatment-resistant brain cancers [141]. These studies have revealed that the gut microbiota composition in patients with glioma differs significantly from that in healthy individuals, suggesting that an imbalanced microbiome may contribute to the pathogenesis and progression of brain tumors [142]. The gut microbiota of glioblastoma patients has been found to exhibit reduced microbial diversity and an overrepresentation of certain bacteria, such as *Firmicutes* and *Proteobacteria*, compared to controls [141]. Likewise, gut dysbiosis in these patients is often accompanied by changes in metabolite profiles, including increased levels of pro-inflammatory cytokines and altered SCFA concentrations [143]. These metabolic shifts can affect systemic immune function, potentially influencing tumor growth and immune evasion mechanisms [144]. Studies have also demonstrated that microbiome-based therapies, such as probiotics or fecal microbiota transplantation (FMT), may have therapeutic potential in glioblastoma patients [145]. For instance, certain probiotics [146] have been shown to enhance the immune response against tumors by promoting anti-tumor immune cell production and improving chemotherapy efficacy [147]. While these results are still preliminary, they suggest that microbiome modulation may be a promising adjunct to traditional cancer treatments for brain tumors. Clinical evidence linking gut dysbiosis with brain tumors flags the critical role of gut-derived metabolites in influencing both local tumor growth and systemic immune responses [148]. Further clinical studies are needed to validate these findings and explore the potential for microbiome-based interventions in treating brain cancers.

### 4.3. Novelty: Limited Longitudinal Human Studies Linking Microbiome Interventions to Neuro-Cancer Outcomes

Despite the growing body of research on the gut microbiota and its relationship to both neurobiology and cancer, there is a significant gap in longitudinal human studies that link microbiome interventions to neuro-cancer outcomes [149]. While preclinical studies and initial clinical trials suggest promising results, long-term data that demonstrate the sustained effects of microbiome-based interventions, such as probiotics, prebiotics, or FMT, on neuro-cancer outcomes are still lacking [150]. One of the significant obstacles in this area is the complexity of human microbiomes and their variability across individuals. Factors such as diet, genetics, lifestyle, and environmental exposures can all influence the composition and function of the microbiome, making it difficult to establish definitive cause-and-effect relationships between microbiome alterations and cancer or neurodegenerative diseases [151]. The long-term effects of microbiome interventions on tumor progression, immune response, and brain health remain poorly understood. To fully realize the therapeutic potential of microbiome-based treatments, there is a pressing need for large-scale, longitudinal human studies that track microbiome alterations over time and assess their direct impact on cancer outcomes, neuroinflammation, and cognitive function [152]. Such studies will not only help clarify the role of the gut microbiota in the brain–cancer axis but also inform the development of targeted, evidence-based microbiome interventions for neurodegenerative diseases and cancer treatment [153]. The novelty of this area lies in the untapped potential for microbiome-based therapies in clinical oncology and neurology. A more profound realization of the long-term effects of microbiome interventions could lead to groundbreaking advances in personalized medicine, offering patients more effective treatments that harness their gut microbiota’s power to fight cancer and neurological diseases [154]. Table 2 overviews the key preclinical and clinical studies exploring the link between gut microbiota, neurobiology, and cancer. The research findings indicate the promise of microbiome-based interventions in cancer and neurological outcomes, but several limitations and research gaps persist. These include the need for more extensive, consistent clinical trials, deeper mechanistic interpretation, and a clearer insight into how specific gut-derived metabolites [155] influence the brain–cancer axis.

## 5. Case Studies: Gut Microbiota Modulation in Neuro-Cancer Interventions

This section delves into a series of case studies that explore the potential for gut microbiota modulation in neuro-cancer interventions. These case studies draw attention to the various clinical and preclinical approaches where microbial interventions, such as probiotics, prebiotics, FMT, and diet modifications, have been tested to influence cancer progression and neurobiological outcomes. Including these case studies provides practical insights into the therapeutic potential of gut microbiota modulation in managing brain tumors and neuroinflammation, bridging the gap between experimental research and clinical applications [159]. These case studies outline the complications and successes in the field and discuss the factors influencing the outcomes of microbiome interventions [160]. These factors include variations in microbial composition among individuals, differences in cancer types, and the complexities of how gut-derived metabolites interact with both the immune system and brain cells. Furthermore, the case studies shed light on the novelty of using gut microbiota manipulation as a potential adjunctive therapy for brain cancer, an area that remains underexplored in clinical practice [161]. Building upon the findings of Zhang et al. (2024), we further elaborate on this aspect by discussing how certain bacteria can penetrate the blood–brain barrier, target hypoxic tumor microenvironments, and activate both innate and adaptive immune responses, thereby presenting promising therapeutic avenues for central nervous system tumors [162]. Plus, we explore the role of engineered bacteria and their derivatives in targeted drug delivery and the modulation of the immune microenvironment through the gut–brain axis. These insights underscore the potential of gut microbiota manipulation in brain cancer therapy and highlight its emerging significance in clinical applications. This section aims to demonstrate the promise of microbiome-based therapies while also noting the gaps and future directions for research in this rapidly emerging field.

### 5.1. Case Studies Showing Dietary Interventions, FMT, and Probiotics in Neuro-Cancer Contexts

The application of microbiome-based interventions, such as dietary changes, FMT, and probiotics, has generated growing interest in neuro-cancer research. These interventions aim to modulate gut microbiota composition and influence systemic processes, including immune responses, inflammation, and tumor progression, which are central to both cancer development and neurobiology [163].

• Dietary interventions in neuro-cancer: Several studies have investigated the effects of nutritional interventions on gut microbiota and their subsequent influence on brain cancer progression [164]. In one case study, patients with brain disorders were placed on a diet rich in fiber and fermented foods. The aim was to promote the growth of beneficial gut bacteria that produce SCFAs, particularly butyrate, which is known for its anti-inflammatory and immune-modulating properties [165]. The results showed that these dietary changes led to an improvement in immune responses and a reduction in systemic inflammation, suggesting a potential pathway for slowing glioblastoma progression [166]. However, this study was limited by a small sample size, and more extended follow-up periods are necessary to assess the long-term efficacy of such dietary interventions [167];
• FMT in brain cancer: FMT, a procedure in which fecal material from a healthy donor is transplanted into the gastrointestinal tract of a patient, has gained attention as a potential tool for restoring microbial diversity and improving health outcomes [168]. A case study involving glioma patients demonstrated that FMT from healthy donors had a positive impact on gut microbial diversity, which was associated with better clinical outcomes in terms of immune modulation and reduced tumor growth [142]. In these patients, FMT was combined with conventional therapies, such as chemotherapy, to enhance treatment efficacy. The issue here lies in the heterogeneity of microbiome composition between individuals and the difficulty of identifying consistent microbial profiles that can be linked to successful outcomes [169];
• Probiotics in neuro-cancer therapy: Probiotics have been tested in various cancer therapies for their ability to restore gut microbiota balance and influence the tumor microenvironment [170]. A case study involving glioma patients undergoing treatment found that supplementation with specific probiotic strains improved immune responses and reduced treatment-related side effects [171]. Furthermore, specific probiotic strains were shown to produce metabolites such as SCFAs, which can inhibit the growth of cancer cells and modulate neuroinflammation [172]. However, these studies also marked the variability in response based on the strain of probiotics, underscoring the need for more research into which probiotics may have specific neuro-cancer therapeutic benefits [173].

### 5.2. Novelty: Spotlighting Failures and Complications in Translating Gut–Brain Interventions to Cancer Therapy

Despite the promising results from preclinical studies and clinical trials investigating gut microbiota modulation in cancer therapy, translating these interventions into widespread clinical practice presents several issues [174]. One significant hurdle is inconsistent, reproducible results across different patient populations and cancer types [175]. Microbial composition is highly individualized, meaning interventions like probiotics or FMT may work effectively for some individuals but fail for others, depending on the patient’s baseline microbiome and genetic factors [176].

• Microbial variability and personalized medicine: The complexity of individual microbiomes and their interaction with genetic and environmental factors complicates the development of standardized microbiome-based therapies for glioma [177]. For example, the variability in gut microbiota among cancer patients, particularly those with brain tumors, may contribute to the differential responses observed in clinical studies [178]. Personalized approaches, which tailor treatments to an individual’s microbiome profile, could help address this complication, but such strategies are still in the early stages of development [179];
• Lack of longitudinal human studies: Another problem is the lack of long-term, longitudinal studies that investigate the sustained effects of microbiome-based interventions on cancer progression and neurobiology [180]. While there have been numerous short-term studies examining the immediate effects of dietary changes, probiotics, and FMT on gut microbiota composition and cancer outcomes, there is a dearth of data on the long-term impact of these interventions [181]. Without longitudinal studies, it is difficult to determine whether these interventions can provide lasting benefits in slowing tumor progression or improving the quality of life for patients with brain cancer [182];
• Regulatory and safety concerns: The safety and regulatory issues surrounding FMT and probiotics are also significant barriers to their widespread adoption in clinical oncology. FMT, while promising, carries the risk of transmitting infections or undesirable microbial species, and its clinical application is still subject to strict regulatory oversight [183]. Similarly, the safety of long-term probiotic use, particularly in immunocompromised cancer patients, remains a concern. Further research into the safety profiles and potential side effects of microbiome interventions is essential before they can be incorporated into routine cancer therapy [184];
• Limited acumen of mechanisms: The lack of grasp of the mechanisms by which gut microbiota-derived metabolites influence both the immune system and cancer progression is a significant obstacle [185]. While metabolites such as SCFAs and tryptophan derivatives have been implicated in immune modulation and tumor suppression, the exact pathways and interactions between these metabolites [155], the gut–brain axis, and the tumor microenvironment remain poorly understood. Further mechanistic studies are needed to elucidate how specific metabolites exert their effects and how these processes can be harnessed for therapeutic purposes. While gut microbiota modulation holds promise as an adjunctive therapy for brain cancer, significant trials remain in standardizing treatments, ensuring the safety and intuition of the underlying biological mechanisms [186]. Table 3 provides an overview of the available literature on gut microbiota-based interventions in neuro-cancer therapies. It highlights the key outcomes and setbacks faced in translating these therapies into standard clinical practice. Further research with larger sample sizes, long-term follow-ups, and standardized protocols is needed to validate these approaches.

## 6. Complications and Knowledge Gaps

Despite promising insights into the gut–brain–cancer axis, significant trials remain in fully knowing and leveraging gut microbiota modulation for cancer therapy. Key knowledge gaps include the lack of comprehensive in vivo studies, limited longitudinal human data, and variability in microbiome interventions [192]. Furthermore, the complexity of microbiota–host interactions, the diversity of microbiome compositions across individuals, and the difficulty in translating preclinical findings to clinical settings pose substantial barriers to advancing neuro-cancer interventions [193]. Approaching these gaps is crucial for optimizing therapeutic strategies and ensuring their efficacy in diverse patient populations.

### 6.1. Complexity of Microbiome–Host Interactions: Variability Among Individuals

One major issue in recognizing the gut–neuro–cancer axis is the inherent variability in microbiome compositions across individuals. Each person’s gut microbiota is shaped by various factors, including diet, genetics, environment, and lifestyle, making it challenging to establish universally applicable interventions [194]. This variability complicates efforts to identify consistent biomarkers or therapeutic targets. The interactions between the microbiome and host systems, including immune responses, metabolic processes, and cancer progression, are highly individualized, further complicating the development of standardized treatments [195].

### 6.2. Limited Mechanistic Studies Linking Specific Metabolites to Neuro-Cancer Pathways

Although there is growing evidence of the role of gut microbiota-derived metabolites in influencing neurobiology and cancer, the mechanistic insight of how specific metabolites modulate neuro-cancer pathways remains limited [196]. Few studies have directly linked metabolites such as short-chain fatty acids, polyamines, and bile acids to specific molecular mechanisms in the brain and tumors [197]. This lack of detailed mechanistic insight hinders the development of targeted interventions that could effectively modulate these pathways for therapeutic benefit [198].

### 6.3. Novelty: Proposing Standardized Multi-Omics Approaches to Tackle These Setbacks

A novel approach is needed to address gut microbiota research’s drawbacks and knowledge gaps. Standardized multi-omics strategies, incorporating genomics, transcriptomics, metabolomics, and proteomics, could provide more perception of the complex interactions between microbiota, the host, and cancer [199]. By integrating these data across diverse populations and experimental models, researchers can identify universal biomarkers, elucidate metabolic pathways, and develop more personalized and effective treatments. Multi-omics approaches hold the potential to unravel the complexities of the gut–brain–cancer axis and bridge the gap between preclinical findings and clinical applications [200].

## 7. Therapeutic Potential and Future Directions

The therapeutic potential of modulating the gut microbiota to influence neurobiology and cancer progression is vast, yet still in its early stages. Future research should focus on optimizing microbiome-based interventions, such as dietary modifications, probiotics, and FMT, to enhance cancer treatment outcomes [201]. Advances in personalized medicine, combined with multi-omics approaches, will be crucial in identifying effective, individualized therapies. Moving forward, more robust clinical trials, mechanistic studies, and long-term data are necessary to translate preclinical successes into viable, safe, and effective therapeutic strategies for cancer patients [202].

### 7.1. Microbiota-Based Interventions: Probiotics, Prebiotics, and Postbiotics

Microbiota-based interventions, such as probiotics, prebiotics, and postbiotics, offer promising strategies to influence the gut–neuro–cancer axis. Probiotics, live beneficial microbes, can modulate gut microbiota composition and enhance immune responses. Prebiotics, dietary fibers that nourish beneficial bacteria, may support long-term microbiome health and provide anti-cancer effects [203]. Postbiotics, bioactive compounds produced by microorganisms, have shown potential in modulating inflammation and promoting anti-tumor immunity. These interventions, individually or in combination, represent a novel approach to improving cancer therapy by targeting gut microbiota [204].

### 7.2. Personalized Approaches: Leveraging Metabolomics and Precision Medicine

Personalized medicine, powered by metabolomics and genomic data, offers a tailored approach to cancer treatment by considering individual microbiome compositions and responses. By leveraging metabolic profiles, clinicians can better understand how a patient’s microbiome influences cancer progression and neurobiology, allowing for more targeted interventions [205]. Precision medicine can optimize the selection of microbiota-based therapies, ensuring they are more effective and specific to each patient’s unique microbiome and tumor characteristics, ultimately improving clinical outcomes.

### 7.3. Novelty: Need for Bi-Directional Research Frameworks Integrating Microbiome and Neuroscience into Cancer Therapy

A bi-directional research framework that integrates microbiome research with neuroscience is crucial for advancing cancer therapy [206]. By examining how gut microbiota affects both neurobiology and cancer progression, this framework enables researchers to identify innovative strategies to modulate these pathways concurrently [207]. The bi-directional nature of this framework emphasizes the reciprocal relationship between the gut microbiota and the brain; gut-derived metabolites influence brain function and neuroinflammation, while the brain can, in turn, impact gut microbiota composition and function through the central nervous system [208]. This reciprocal interaction creates a continuous feedback loop, wherein alterations in one system can affect the other, contributing to cancer progression and therapy responses [209]. Understanding the microbiome’s influence on brain function and tumor behavior will lead to more effective therapeutic approaches for addressing neuroinflammation and tumor growth in cancer treatment. This integrated approach illustrates the importance of connecting the gut–brain axis in developing comprehensive cancer therapies [186].

## 8. Conclusions

The interplay between gut microbiota metabolites, neurobiology, and cancer represents a complex and emerging area of research with significant therapeutic potential. Gut microbiota-derived metabolites, such as short-chain fatty acids, tryptophan derivatives, and bile acids, play crucial roles in modulating immune responses, neuroinflammation, and tumor progression. However, key knowledge gaps remain, particularly regarding the mechanisms underlying these interactions and the translation of preclinical findings into clinical therapies. Closing these research gaps through multi-omics approaches and bi-directional frameworks that integrate microbiome and neuroscience with cancer therapy is essential for developing targeted, personalized interventions. Exploring microbiota-based therapies like probiotics, prebiotics, and postbiotics could provide novel avenues for improving cancer treatment outcomes and patient quality of life.

## Figures and Tables

**Table 1:** Comparative Analysis of Gut Microbiota-Derived Metabolites and Their Known Effects on Neurobiology and Cancer Pathways.

Metabolite	Gut Microbiota Genera	Neurobiological Effects	Cancer Pathway Effects	Knowledge Gaps and Research Opportunities
SCFAs	Bacteroides, *Lactobacillus*, *Clostridium*	Improve blood–brain barrier (BBB) integrity; modulate neurotransmitter release; and reduce neuroinflammation	Suppress tumor growth via apoptosis; regulate immune checkpoints; and inhibit angiogenesis	Mechanisms linking SCFAs to both neuroprotection and cancer suppression remain underexplored [7]
Tryptophan Metabolites	*Lactobacillus*, *Clostridium*	Modulate serotonin pathways; interact with the AhR for anti-inflammatory effects	Influence estrogen metabolism; exhibit anti-tumor activity through AhR-mediated pathways	Role in the neuro–cancer connection and their therapeutic potential require deeper investigation [84]
Secondary Bile Acids	Bacteroides, *Clostridium*, *Enterococcus*	Reduce neuroinflammation via farnesoid X receptor (FXR) signaling; enhance neuronal protection	Promote or suppress carcinogenesis depending on context; influence DNA damage and repair mechanisms	Dual roles in neuroprotection and oncogenesis are poorly understood [85]
LPS	*Escherichia coli*, *Salmonella*	Trigger neuroinflammation via microglial activation; impair BBB function	Drive tumor-promoting inflammation; inhibit anti-tumor immunity	How chronic low-grade LPS exposure links gut, brain, and cancer remains unclear [86]
Indoles	*Clostridium*, *Escherichia coli*	Enhance BBB function; reduce neuroinflammation through AhR signaling	Induce apoptosis in cancer cells; modulate estrogen metabolism	Limited acumen of their systemic signaling pathways in the neuro–cancer axis [87]
Polyamines	*Enterobacteriaceae*, *Bifidobacterium*	Neuroprotective effects via autophagy modulation; regulate synaptic plasticity	Support tumor growth by promoting angiogenesis and cellular proliferation; potential anti-tumor effects in specific contexts	Context-dependent roles in neuroprotection versus cancer promotion require detailed examination [88]
Emerging Metabolites (e.g., Phenolics, Sphingolipids)	Firmicutes, Bacteroides	Potential to modulate oxidative stress and mitochondrial function in neurons	Understudied effects on DNA repair and immune modulation in cancer	Novel mechanisms connecting these metabolites to the gut–brain–cancer axis remain largely unexplored [89]

**Table 2:** Summary of Key Preclinical and Clinical Studies, Including Findings, Limitations, and Research Gaps.

Study Type	Study Focus/Objective	Key Findings	Limitations	Research Gaps
Preclinical	Rodent models of gut dysbiosis and cancer	Gut dysbiosis is linked to neuroinflammation and tumor progression. Gut-derived SCFAs (e.g., butyrate) are shown to modulate immune responses and tumor microenvironment.	Limited to rodent models; cannot directly translate to humans. Variability in results based on rodent strain.	In-depth exploration of how specific microbial taxa contribute to neuro-cancer progression. Lack of mechanistic concept of how SCFAs and other metabolites regulate brain–cancer cross-talk.
Preclinical	Rodent models of gut microbiota modulation in neurobehavioral changes	Dysbiosis leads to altered behaviors (e.g., anxiety and depression). Gut microbiota affects blood–brain barrier permeability and neuroinflammation.	Results are dependent on specific microbiota interventions. Lack of longitudinal studies in rodents.	Need for in vivo studies focusing on the long-term effects of gut microbiota interventions on brain function. Investigating the role of gut-derived metabolites in neurobehavioral changes [156].
Clinical	Clinical trials on gut microbiota and glioma	Altered microbiome composition found in glioma patients, including decreased microbial diversity. Probiotics and FMT show potential for improving immune response in cancer patients.	Small sample sizes. Variability in patient microbiomes.	Larger, randomized controlled trials are needed. Studies on microbiome interventions as adjunctive therapies in glioma treatment [142].
Clinical	Studies on microbiome alterations in gliobastoma patients	Dysbiosis observed in patients with brain cancers. Correlation between specific microbiota profiles and poor prognosis in brain cancer.	Lack of consistency in study results. Limited studies on microbiome and brain cancer specifically.	Further studies needed on the role of gut-derived metabolites in brain cancer progression and therapy. More robust clinical data linking gut microbiota to glioblastoma outcomes [157].
Clinical	Human trials on probiotics and prebiotics in cancer patients	Some improvement in immune response and tumor progression in early-phase trials with probiotics.	Early-phase trials with small cohorts. Long-term outcomes and effects on overall survival are not established.	Longitudinal, large-scale trials examining the long-term effects of microbiome-based interventions in cancer treatment. Clarifying the mechanisms by which microbiome modulation impacts cancer and neurodegenerative diseases [158].

**Table 3:** Case studies on gut microbiota modulation in neuro-cancer interventions, including the type of intervention, key outcomes, and limitations.

Study	Intervention Type	Outcomes	Limitations
Dietary Intervention in Brain Cancer Patients	High-fiber and fermented food diet	Increased production of SCFAs (e.g., butyrate); improved immune responses; and reduced systemic inflammation	Small sample size; short duration; and no control group [187]
FMT in Glioma Patients	FMT from healthy donors	Improved gut microbiota diversity; enhanced immune responses; and reduced tumor growth	Microbial variability among individuals; no long-term follow-up data; and heterogeneity in clinical outcomes [188]
Probiotics in Brain Cancer Patients	Probiotic supplementation (specific strains)	Reduction in treatment-related side effects; enhanced immune function; and increased SCFA production	Variability in probiotic strains; inconsistent response among patients; and limited long-term data [189]
Dietary and Probiotic Intervention in Brain Cancer	Combined dietary changes (fiber-rich diet) and probiotic supplementation	Decreased neuroinflammation; improved tumor response in preclinical models	Animal model-based study; limited human data; and lack of standardized probiotic strain [190]
FMT in Combination with Chemotherapy in Cancers	FMT combined with conventional chemotherapy	Increased efficacy of chemotherapy; altered tumor microenvironment; and improved gut health	Small patient cohort; potential safety concerns in immunocompromised patients; and no detailed microbial profiling [191]

## Data Availability

No new data were created or analyzed in this study. Data sharing is not applicable to this article.

## Abbreviations

The following abbreviations are used in this manuscript:

SCFAs	Short-chain fatty acids
LPS	Lipopolysaccharides
GBA	Gut–brain axis
CNS	Central nervous system
ENS	Enteric nervous system
AhR	Aryl hydrocarbon receptor
I3C	Indole-3-carbinol
DNA	Deoxyribonucleic acid
BBB	Blood–brain barrier
FXR	Farnesoid X receptor
HDACs	Histone deacetylases
TNF-α	Tumor necrosis factor-alpha
IL-6	Interleukin-6
FMT	Fecal microbiota transplantation
